# The impact of clomiphene citrate on the endometrium in comparison to gonadotropins in intrauterine insemination cycles: is it thinner and does it matter?

**DOI:** 10.3389/fendo.2024.1414481

**Published:** 2024-06-24

**Authors:** Yao Lu, Panagiotis Cherouveim, Victoria Jiang, Irene Dimitriadis, Kaitlyn E. James, Charles Bormann, Irene Souter

**Affiliations:** ^1^ Massachusetts General Hospital Fertility Center, Department of Obstetrics, Gynecology, and Reproductive Biology, Massachusetts General Hospital and Harvard Medical School, Boston, MA, United States; ^2^ Department of Reproductive Medicine, Ren Ji Hospital, Shanghai Jiao Tong University School of Medicine, Shanghai, China; ^3^ Shanghai Key Laboratory for Assisted Reproduction and Reproductive Genetics, Shanghai, China

**Keywords:** endometrial thickness, ovarian stimulation, clomiphene, gonadotropin, intrauterine insemination

## Abstract

**Objective:**

To determine whether endometrial thickness (EMT) differs between i) clomiphene citrate (CC) and gonadotropin (Gn) utilizing patients as their own controls, and ii) patients who conceived with CC and those who did not. Furthermore, to investigate the association between late-follicular EMT and pregnancy outcomes, in CC and Gn cycles.

**Methods:**

Retrospective study. Three sets of analyses were conducted separately for the purpose of this study. In analysis 1, we included all cycles from women who initially underwent CC/IUI (CC1, n=1252), followed by Gn/IUI (Gn1, n=1307), to compare EMT differences between CC/IUI and Gn/IUI, utilizing women as their own controls. In analysis 2, we included all CC/IUI cycles (CC2, n=686) from women who eventually conceived with CC during the same study period, to evaluate EMT differences between patients who conceived with CC (CC2) and those who did not (CC1). In analysis 3, pregnancy outcomes among different EMT quartiles were evaluated in CC/IUI and Gn/IUI cycles, separately, to investigate the potential association between EMT and pregnancy outcomes.

**Results:**

In analysis 1, when CC1 was compared to Gn1 cycles, EMT was noted to be significantly thinner [Median (IQR): 6.8 (5.5–8.0) vs. 8.3 (7.0–10.0) mm, p<0.001]. Within-patient, CC1 compared to Gn1 EMT was on average 1.7mm thinner. Generalized linear mixed models, adjusted for confounders, revealed similar results (coefficient: 1.69, 95% CI: 1.52–1.85, CC1 as ref.). In analysis 2, CC1 was compared to CC2 EMT, the former being thinner both before [Median (IQR): 6.8 (5.5–8.0) vs. 7.2 (6.0–8.9) mm, p<0.001] and after adjustment (coefficient: 0.59, 95%CI: 0.34–0.85, CC1 as ref.). In analysis 3, clinical pregnancy rates (CPRs) and ongoing pregnancy rates (OPRs) improved as EMT quartiles increased (Q1 to Q4) among CC cycles (p<0.001, p<0.001, respectively), while no such trend was observed among Gn cycles (p=0.94, p=0.68, respectively). Generalized estimating equations models, adjusted for confounders, suggested that EMT was positively associated with CPR and OPR in CC cycles, but not in Gn cycles.

**Conclusions:**

Within-patient, CC generally resulted in thinner EMT compared to Gn. Thinner endometrium was associated with decreased OPR in CC cycles, while no such association was detected in Gn cycles.

## 1 Introduction

Infertility affects 8–15% of reproductive age couples and has become a global health issue (1, 2). Treatments such as ovarian stimulation (OS) with intrauterine insemination (IUI) are simpler, and less expensive than *in-vitro* fertilization (IVF). Therefore, OS/IUI is often the recommended first-line treatment for couples with unexplained, ovulatory, and mild male factor infertility (3, 4). As a matter of fact, over 155,000 IUI cycles are performed each year in Europe alone, according to data from the European Society of Human Reproduction and Embryology (ESHRE) (5, 6).

Clomiphene citrate (CC) and gonadotropins (Gn) are frequently used for OS/IUI treatments (7). Both medications, through different mechanisms of action, promote follicular growth. However, CC, often used in OS as the first line medication, also has estrogen antagonistic properties on the endometrium, eventually affecting its growth and potentially the ability of an embryo to implant in it (8). Similarly, Gn by stimulating multi-follicular growth, increase estrogens to, on occasion, supraphysiologic levels, potentially impacting endometrial development as well as receptivity (8, 9).

Yet, studies evaluating the effect of the different OS regimens on endometrial thickness (EMT) and the potential association of the latter with pregnancy outcomes in IUI cycles have been limited and inconclusive. A few authors reported that the endometrium was thinner in CC compared to Gn-stimulated cycles (10), while others did not find a difference (11, 12). In addition, there have been data suggesting that a thinner endometrium might be associated with decreased chances of pregnancy in both CC and Gn cycles (13–15), and some earlier studies reported no pregnancies with EMT lower than certain cut-offs (14, 16). However, ultrasound technology has improved dramatically since the latter studies potentially permitting a more precise measurement of the endometrium. A recent retrospective study of 1065 Gn cycles showed that the pregnancy rate was the highest when EMT was in the range of 10.5–13.9 mm, and lowest when EMT was less than 7 mm (17). On the contrary, data from a secondary analysis of the Assessment of Multiple Intrauterine Gestations from Ovarian Stimulation (AMIGOS) randomized controlled trial (RCT) showed that although EMT was thinner in CC, as compared to Gn cycles, among patients with unexplained infertility, pregnancy rates were not associated with EMT in either group (18). Results from a meta-analysis including various OS regimens also found no evidence of an association between EMT and IUI outcomes (4). The existing studies, albeit compelling, have their own limitations, including either a small sample size, or a focus on a specific infertility diagnosis.

Currently, it remains unclear whether, in the same patient, OS with CC produces a late-follicular endometrium that is thinner than that of Gn-stimulated cycles. Furthermore, it is uncertain whether such differences have a consequential effect on OS/IUI pregnancy outcomes. The present study aimed to determine whether EMT differs between CC/IUI and Gn/IUI cycles primarily by means of utilizing patients as their own controls. Furthermore, we aspired to investigate the potential association, if any, between late-follicular EMT and pregnancy outcomes (namely clinical pregnancy, spontaneous abortion, and ongoing pregnancy) among different OS regimens in IUI cycles.

## 2 Materials and methods

### 2.1 Study population and design

This retrospective study was approved by Partners Institutional Review Board. Data from 15980 cycles of 4783 women undergoing IUI between January 2004 and September 2021 at the Massachusetts General Hospital (MGH) Fertility Center were reviewed for eligibility (**Supplementary Figure 1**). Exclusion criteria included the diagnosis of uterine factor infertility, and/or severe tubal/peritoneal factor with co-existing, untreated hydrosalpinges. Cycles with no available EMT information at the time of the last ultrasonographic evaluation were also excluded.

**Figure 1:**
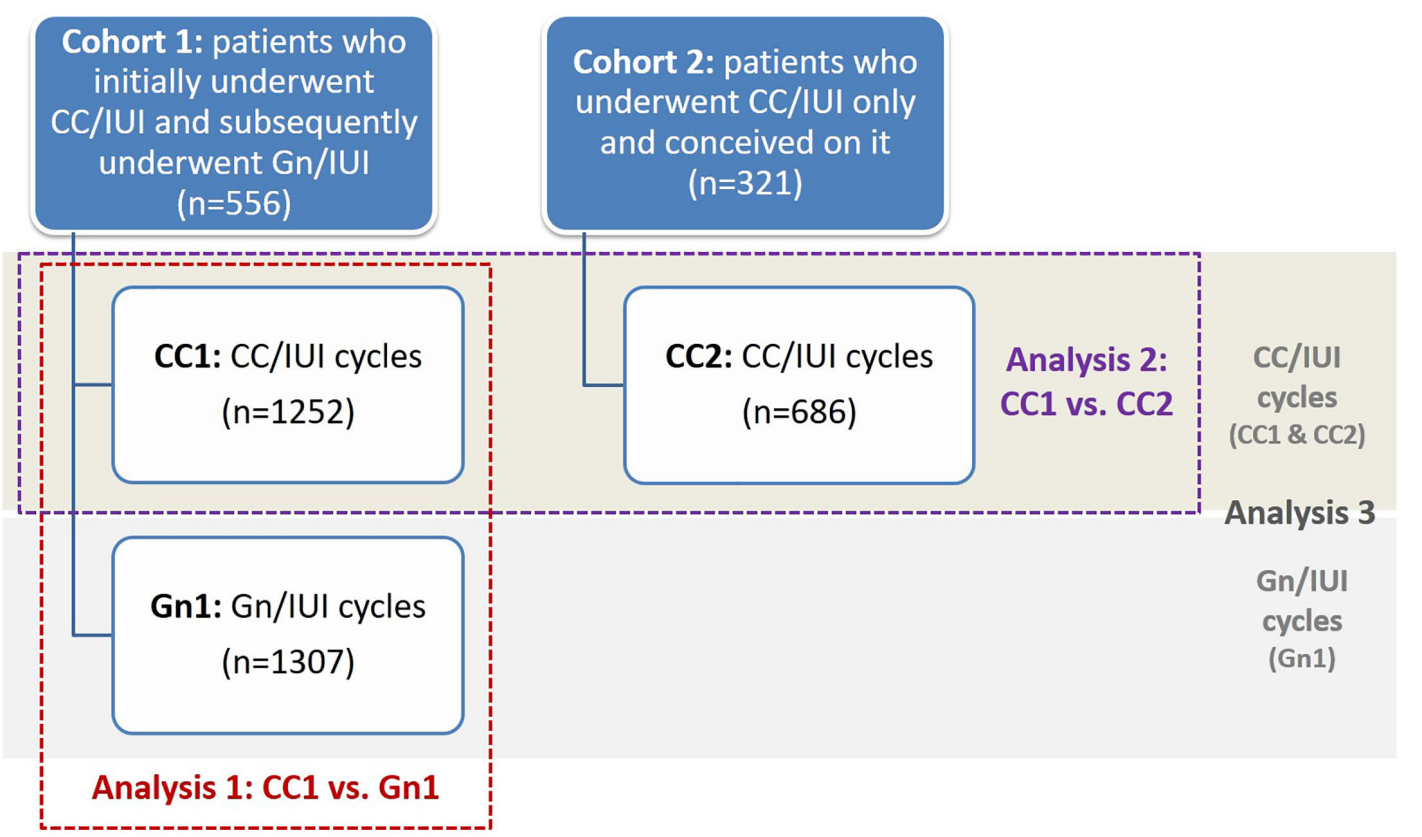
Study design. CC, clomiphene; EMT, endometrial thickness; Gn, gonadotropin; IUI, intrauterine insemination. Analysis 1: EMT comparison between CC1 and Gn1, utilizing women as their own controls. The purpose of analysis 1 was to answer: Does EMT differ in CC compared to Gn cycles, in the same patient? Analysis 2: EMT comparison between CC1 and CC2. The purpose of analysis 2 was to answer: Does EMT differ between CC cycles leading to conception and those not? Analysis 3: Association between pregnancy outcomes and EMT quartiles in CC/IUI, and Gn IUI cycles, separately. CC cycles included all cycles in CC1 and CC2. The purpose of analysis 3 was to answer: Is there an association between EMT and pregnancy outcomes among different OS regimens?

As shown in **Figure 1**, for the purpose of this study, two cohorts of women were included, and three sets of analyses were conducted separately.

In analysis 1, we sought to evaluate CC’s impact on the endometrium in comparison to Gn, utilizing women as their own controls. For this purpose, we included in cohort 1, all cycles from women who sought fertility treatments undergoing initially CC/IUI (CC1, n=1252), followed by Gn/IUI (Gn1, n=1307), and compared CC1 to Gn1 cycles. The purpose of analysis 1 was to answer the following question: Does EMT differ in CC compared to Gn cycles in the same patient?

In analysis 2, we aimed to evaluate potential EMT differences between patients who conceived with CC and those who did not. For this purpose, we included all the CC/IUI cycles from women who eventually conceived with CC during the same study period (CC2, n=686) in study cohort 2, and compared the EMT between CC2 and CC1 cycles. The purpose of analysis 2 was to answer the following question: Does EMT differ between CC cycles leading to conception and those not?

Furthermore, a 3^rd^ analysis was performed and pregnancy outcomes [clinical pregnancy, spontaneous abortion, and ongoing pregnancy rates (CPR, SABR, and OPR, respectively)] among different EMT quartiles were evaluated in CC/IUI, and Gn/IUI cycles, separately. The goal of the 3^rd^ analysis was to answer the following question: Is there an association between EMT and pregnancy outcomes among different OS regimens (CC and Gn)?

### 2.2 IUI protocols

All couples had completed a standard infertility evaluation prior to treatment initiation as previously reported (19). Briefly, all women undergoing OS/IUI had at least one patent fallopian tube and partner’s sperm had post processing total motile sperm count ≥ 1 million. All patients included in the study underwent at least one monitored CC/IUI cycle. However, women in cohort 1, after failing CC/IUI attempts (CC1), eventually pursued OS/IUI utilizing gonadotropins (Gn1), while women in cohort 2 achieved pregnancy with CC/IUI treatments (CC2) and did not require gonadotropins.

The standard starting CC dose was 50 mg, with instructions to take it for 5 days starting on cycle days 2 through 5 after spontaneous menses or a progestin-induced withdrawal bleeding. Response to CC was monitored by serial transvaginal ultrasonography and monitoring frequency was individualized after mid-follicular phase. Ovulation was triggered with recombinant HCG (Ovidrel, Merck Serono), when at least one dominant follicle reached 16 mm in diameter. CC dose was increased to 100 mg or 150 mg in subsequent cycles either for the indication of no response to the previously administered dose or at physicians’ recommendation (usually to increase follicular response). In the rare situation, where patients had an exaggerated follicular response to 50 mg, the dose was decreased to 25 mg in subsequent cycles.

Patients not conceiving with CC/IUI eventually were advanced to Gn/IUI treatments and initiated recombinant follicle stimulating hormone (rFSH) on cycle day 3. Starting dose was individualized based on age, body mass index (BMI), ovarian reserve biomarkers, and prior response. Follicular development in Gn cycles was monitored by serial transvaginal ultrasonography and serum estradiol (E_2_) levels. FSH dose was adjusted, as needed, to achieve follicular response. Ovulation was triggered with Ovidrel when at least one lead follicle reached 16 mm in largest diameter.

Single IUI was performed 35–36 hours after HCG-trigger with either donor or washed partner’s sperm by a trained health care professional.

A pregnancy test was performed approximately two weeks after the IUI, with a serum β-HCG level over 6 mIU/mL considered positive. Clinical pregnancy was confirmed, via transvaginal ultrasonography, with the detection of at least one gestational sac at approximately 6 weeks of pregnancy. Spontaneous abortion (SAB) was defined as the loss of a previous sonographically-confirmed clinical pregnancy. Ongoing pregnancy was defined as an intrauterine pregnancy with a sonographically-confirmed fetal heartbeat at the time the woman was discharged to the obstetrics service for prenatal follow-up, usually around 8 weeks of gestation.

### 2.3 Outcome measures

The primary outcome was EMT, as measured and recorded on the last ultrasound (UTZ) before HCG-trigger (the last UTZ was performed for the most part on the day of HCG-trigger, while the remaining UTZs were performed either one or two days prior to it). All UTZs were performed by trained health care professionals per routine clinical care. In analysis 1, patients were used as their own controls and EMT was compared between CC1 and Gn1 cycles. In analysis 2, EMT was compared between CC1 and CC2 cycles. Furthermore, to investigate the potential association, if any, between EMT and pregnancy outcomes among different OS regimens, in analysis 3, we evaluated pregnancy outcomes (clinical pregnancy, spontaneous abortion, and ongoing pregnancy rates) among different EMT quartiles for CC/IUI and Gn/IUI cycles, separately (**Figure 1**). For the calculation of CC EMT quartiles, all CC cycles (CC1 and CC2) were included irrespective of conception outcome.

### 2.4 Statistical analysis

Normally distributed continuous variables were expressed as mean ± standard deviation (SD), while non-normally distributed continuous variables as median and interquartile range (IQR). Student’s t-test or Mann-Whitney U test were used, as appropriate. Categorical variables were summarized as frequency (n) and percentage (%), and chi-square test or Fisher’s exact test were used, as appropriate.

Since the last UTZ was performed on cycle days that varied between cycles, analysis was stratified according to the day of last UTZ, where appropriate. Of note, almost half (47.2%) of the late-follicular EMT measurements were taken on the day of HCG-trigger, while approximately one third (36.5%), and one eighth (12.6%) were measured either one or two days prior to HCG-trigger, respectively.

In analysis 1, to estimate the within-patient variability of EMT between CC1 and Gn1 cycles, CC and Gn cycles from the same patient were matched by day of last UTZ (i.e.: EMT measured on the same day in relation to HCG-trigger), and the absolute difference of EMT was calculated among each matched cycle pair. In addition, to account for multiple cycles from the same patient, while controlling for potential confounders, generalized linear mixed models (GLMM) were utilized to estimate potential EMT differences in cohort 1 (CC1 vs. Gn1).

The same analytic approach was also utilized in analysis 2, to estimate the EMT difference between CC1 and CC2. Results were expressed as coefficient (*coeff*.) and 95% confidence interval (CI).

Furthermore, generalized estimating equations (GEE) logistic regression models were implemented to investigate the association between EMT and pregnancy outcomes in CC and Gn cycles, separately. In this case, all CC cycles (whether from CC1 or CC2) were pooled together. EMT was assessed either as a continuous variable or by quartile increment (Quartiles 1–4: Q1-Q4). Results were expressed as odds ratio (OR) and 95% CI. Models were adjusted for potential confounders (including age, BMI, prior gravidity, diagnosis, and day of last UTZ).

A two-sided alpha level of 0.05 was considered statistically significant. SPSS 21.0 (SPSS Inc.) was used for all statistical analyses.

## 3 Results

### 3.1 General data

In study cohort 1, we included a total of 2559 cycles from 556 women, who initially underwent 1252 CC/IUI (CC1). Given lack of success with CC, all women were further advanced to Gn/IUI treatments completing a total of 1307 Gn/IUI cycles (Gn1).

In study cohort 2, a total of 686 CC/IUI cycles (CC2) from 321 women that eventually conceived with CC treatments were included and were compared to CC1 cycles in regards to EMT.

Characteristics of the study population are summarized in **Table 1**. The majority of women were Caucasian (77.3%), and the most common diagnosis was unexplained infertility. Overall, most patients (almost 90%) that conceived with CC did so within 1–3 cycles, and patients who didn’t conceive after a finite number of CC cycles (usually 3 cycles) were either advised to proceed with Gn or IVF, when applicable.

**Table 1:** Baseline characteristics (per patient); Cycle characteristics (per cycle).

a. Baseline characteristics	CC1/Gn1		CC2	p-value	
No. of patients	556		321		
Age (years)	33.5 ± 4.1		32.9 ± 3.5	0.01	
Body mass index (kg/m^2^)	23.1 (21.0–26.3)		23.7 (21.6–27.9)	0.02	
Basal FSH (IU/L)	7.0 ± 2.3		6.7 ± 2.2	0.13	
Prior gravidity n (%)	186 (33.5)		135 (42.1)	0.01	
Prior parity n (%)	97 (17.4)		74 (23.1)	0.05	
Diagnosis n (%)				0.22	
Unexplained	226 (40.6)		120 (37.4)		
Polycystic ovary syndrome	80 (14.4)		63 (19.6)		
Other ovulatory dysfunction	44 (7.9)		25 (7.8)		
Male factor	69 (12.5)		40 (12.5)		
Diminished ovarian reserve	31 (5.6)		8 (2.5)		
Tubal/Peritoneal factor	14 (2.5)		6 (1.9)		
Combined factors	71 (12.8)		41 (12.8)		
Single parent by choice/ Same sex	18 (3.2)		14 (4.4)		
Other	3 (0.5)		4 (1.2)		
b. Cycle characteristics	CC1	Gn1	CC2	p-value	
				CC1 vs. Gn1	CC1 vs. CC2
No. of cycles	1252	1307	686		
Ovarian response					
No. of follicles ≥15mm	1.0 (1.0–2.0)	1.0 (1.0–1.0)	1.0 (1.0–2.0)	<0.001	0.25
No. of follicles ≥13mm	2.0 (1.0–2.0)	2.0 (1.0–2.0)	2.0 (1.0–2.0)	0.004	0.35
Cycle trigger day	12.0 (11.0–14.0)	11.0 (9.0–13.0)	12.0 (11.0–14.0)	<0.001	<0.001
Day of last ultrasound				<0.001	<0.001
Day of HCG-trigger	662 (52.9)	547 (41.9)	337 (49.1)		
One-day prior to HCG-trigger	319 (25.5)	614 (47.0)	230 (33.5)		
Two-days prior to HCG-trigger	216 (17.3)	107 (8.2)	107 (15.6)		
Endometrial thickness					
Overall	6.8 (5.5–8.0)	8.3 (7.0–10.0)	7.2 (6.0–8.9)	<0.001	<0.001
Day of HCG-trigger	7.0 (5.7–8.3)	8.9 (7.4–10.0)	7.5 (6.2–9.0)	<0.001	<0.001
One-day prior to HCG-trigger	6.5 (5.5–8.0)	8.0 (7.0–9.9)	7.1 (6.0–8.7)	<0.001	<0.001
Two-days prior to HCG-trigger	6.5 (5.5–7.9)	8.0 (7.0–9.4)	7.0 (5.7–8.2)	<0.001	0.07

Data are shown as mean ± standard deviation (SD) if normally distributed or median and interquartile range (IQR: 25th–75th) if non-normally distributed or number (percentage).

CC, clomiphene; Gn, gonadotropin; FSH, follicle stimulating hormone.

### 3.2 Analysis 1: EMT differences between CC/IUI and Gn/IUI within the same patient

**Figure 2:**
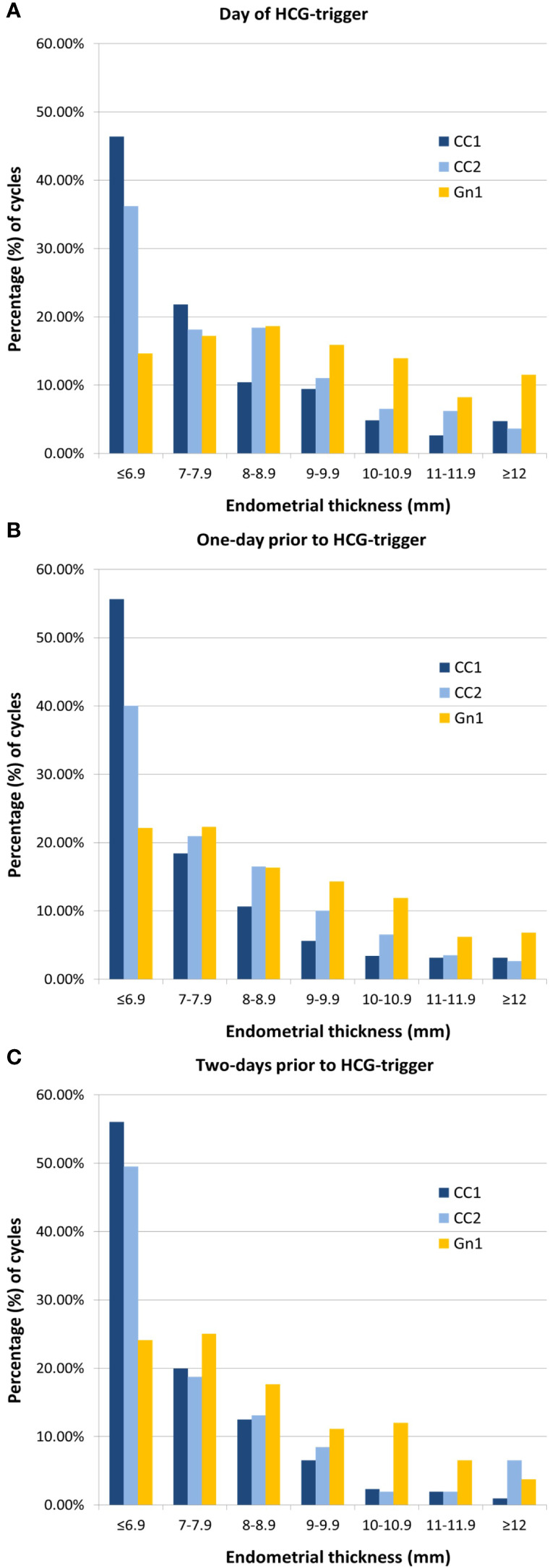
Distribution of endometrial thickness stratified by day of last ultrasound. **(A)** On the day of HCG-trigger. **(B)** One-day prior to HCG-trigger. **(C)** Two-days prior to HCG-trigger. CC, clomiphene; Gn, gonadotropin.

In study cohort 1, follicular response, as assessed by total number of follicles ≥ 13 mm, did not differ clinically between CC1 and Gn1 cycles. However, HCG-trigger was on average one day later in CC1 compared to Gn1 cycles. Despite longer duration of the follicular phase in CC1 cycles, and clinically comparable follicular response, EMT in CC1 cycles was significantly thinner than that of Gn1 (6.8 vs. 8.3 mm, for CC1 vs. Gn1, p<0.001), a finding that was consistent and independent of the day of last UTZ (**Table 1**, **Figures 2A-C**). In addition, 46.4% of CC1 cycles had an EMT < 7 mm on the day of HCG-trigger, while in Gn1 cycles only 14.6% were below the same cut-off (**Figure 2A**). A similar EMT distribution was noted for UTZs performed one or two days prior to HCG-trigger (**Figures 2B, C**).

Subsequently, CC1 cycles from the same patient were matched to Gn1 cycles based on day of last UTZ. Among the 556 patients in study cohort 1, N_0_ = 259, N_1_ = 121, and N_2_ = 16 CC1/Gn1 cycle matches were created based on timing of last UTZ in relation to HCG-trigger that were on the day of, one day, or two days prior to it, respectively. Mean ± SD EMT difference between Gn1 and CC1 was 1.7 ± 2.1 mm [median (IQR): 1.6 (0.5, 3.0)]. More specifically, EMT differences between Gn1 and CC1 cycles were 1.8 ± 2.2 mm [median (IQR): 2.0 (0.5, 3.2)], 1.4 ± 2.0 mm [median (IQR): 1.5 (0.4–2.7)], and 1.2 ± 1.8 mm [median (IQR): 0.6 (-0.1, 1.4)] for UTZs performed on day of, one-day prior, and two-days prior to HCG-trigger, respectively.

Furthermore, a GLMM model was applied in study cohort 1 to account for multiple cycles from the same patient adjusting for age, BMI, prior gravidity, and day of last UTZ (**Table 2**). Overall, EMT in CC1 was significantly thinner as compared to Gn1 by 1.69 mm (*coeff*.: 1.69, 95% CI: 1.52–1.85, p<0.001).

**Table 2:** Multivariate regression models for endometrial thickness.

EMT	Crude			Adjusted*		
	Coefficient	95% CI	p-value	Coefficient	95% CI	p-value
Analysis 1: CC1 vs. Gn1						
CC1	*Ref.*			*Ref.*		
Gn1	1.67	1.51–1.83	<0.001	1.69	1.52–1.85	<0.001
Analysis 2: CC1 vs. CC2						
CC1	*Ref.*			*Ref.*		
CC2	0.67	0.41–0.93	<0.001	0.59	0.34–0.85	<0.001

CC, clomiphene; CI, confidence interval; EMT, endometrial thickness; Gn, gonadotropin; OR, odds ratio.

Generalized linear mixed models were applied.

*Adjusted for age, body mass index, prior gravidity and day of last ultrasound.

### 3.3 Analysis 2: EMT differences between those who conceived with CC and those who did not

CC1 women, when compared to CC2, were older, with both a lower BMI and gravidity/parity. Overall, EMT in CC1 cycles was significantly thinner than that of CC2 (6.8 vs. 7.2 mm, for CC1 vs. CC2, p<0.001), a finding that was independent of the day of the last UTZ. Unlike CC1 cycles, where 46.4% of the cycles had an EMT < 7 mm on the day of HCG-trigger, less CC2 cycles (36.2%) had an EMT below the same cut-off (**Figure 2A**). GLMM models adjusted for age, BMI, prior gravidity, and day of last UTZ suggested that CC1 EMT was 0.59 mm thinner than CC2 cycles (*coeff.:* 0.59, 95% CI: 0.34–0.85, p<0.001).

### 3.4 Analysis 3: association between EMT and pregnancy outcomes in CC and Gn cycles

**Figure 3:**
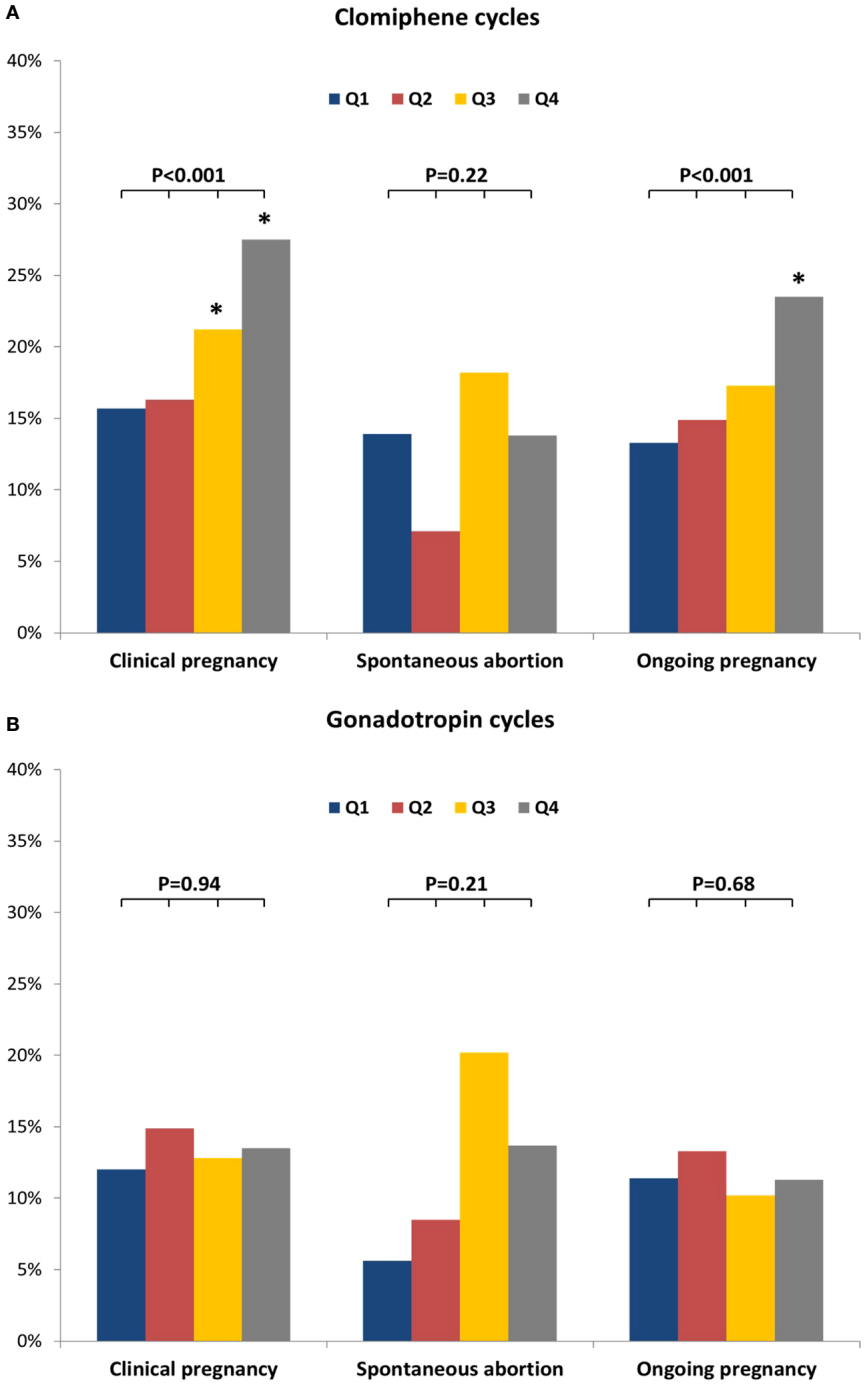
Pregnancy outcomes according to quartiles of endometrial thickness. **(A)** Clomiphene cycles. **(B)** Gonadotropin cycles. Q1- Q4 represents quartiles of endometrial thickness. * represents p<0.05 compared to Q1.

Pregnancy outcomes were compared amongst EMT quartiles (Q1-Q4) in CC and Gn cycles, separately (**Figures 3A, B**). EMT for CC cycles was Q1: ≤ 5.7 mm, Q2: 5.8–6.9 mm, Q3: 7.0–8.1 mm, and Q4: 8.2 mm; while EMT for Gn cycles was Q1: ≤ 6.9 mm, Q2: 7.0–8.2 mm, Q3: 8.3–9.9 mm, and Q4: ≥ 10.0 mm.

Among CC cycles, CPRs improved as EMT quartiles increased (Q1: 15.7%, Q2: 16.3%, Q3: 21.2%, Q4: 27.5%, p<0.001 for all comparisons), while SABRs were similar amongst the different EMT quartiles (Q1: 13.9%, Q2: 7.1%, Q3: 18.2%, and Q4: 13.8%, p=0.22 for all comparisons). OPRs in CC cycles also improved as EMT quartiles increased (Q1: 13.3%, Q2: 14.9%, Q3: 17.3%, and Q4: 23.5%, p<0.001 for all comparisons).

Interestingly, in Gn cycles, CPRs, SABRs and OPRs were all comparable amongst different quartile groups (for Q1-Q4, respectively, CPR were: 12.0%, 14.9%, 12.8%, and 13.5%, p=0.94 for all comparisons; SABRs were: 5.6%, 8.5%, 20.0%, and 13.7%, p=0.21 for all comparisons; and OPR were 11.4%, 13.3%, 10.2%, and 11.3%, p=0.68 for all comparisons).

Additionally, although most pregnancies were observed in cycles with EMT ≥ 25^th^ pct., clinical pregnancies were seen even with an EMT < 5^th^ pct. for both CC and Gn cycles (5^th^ pct. cut-offs: 4.5, and 6 mm on the day of HCG-trigger, for CC and Gn, respectively). Among CC cycles, CPR <5^th^ pct. was significantly lower than those observed ≥5^th^ pct. (4.0% vs. 20.5%, for < 5^th^ and ≥ 5^th^ pct., respectively, p=0.003). We noted no such difference in CPR among Gn cycles (9.5% vs. 13.4%, for < 5^th^ and ≥ 5^th^ pct., respectively, p=0.59).

GEE models adjusted for age, BMI, prior gravidity, diagnosis, and day of last UTZ suggested that in CC cycles, EMT (assessed as a continuous variable) was positively associated with CPR and OPR (CPR: adjOR 1.12, 95% CI 1.07–1.17, p<0.001; OPR: adjOR 1.10, 95% CI 1.04–1.16, p<0.001) (**Table 3**). The odds of clinical pregnancy were significantly increased in EMT Q3 and Q4, compared to Q1 [adjOR (95% CI): 1.45, (1.05–2.01), p=0.02; 2.04 (1.49–2.80), p<0.001; for Q3, and Q4 vs. Q1, respectively), and cycles in EMT Q4 had 2.04 times the odds of resulting in clinical pregnancy compared to those in Q1. Additionally, the odds of ongoing pregnancy were significantly increased in EMT Q4 compared to Q1 [adjOR (95% CI): 1.98 (1.41–2.79), p<0.001), and cycles in EMT Q4 had 1.98 times the odds of resulting in clinical pregnancy compared to those in Q1. On the contrary, no significant associations with CPR or OPR were observed in Gn cycles, neither when EMT was analyzed as a continuous variable nor as quartiles.

**Table 3:** Multivariate regression models for clinical pregnancy and ongoing pregnancy.

	Crude			Adjusted*		
	OR	95% CI	p-value	OR	95% CI	p-value
Clinical pregnancy						
CC cycles ^†^						
EMT	1.12	1.07–1.17	<0.001	1.12	1.07–1.17	<0.001
Q1	*Ref.*			Ref.		
Q2	1.05	0.74–1.49	0.79	1.06	0.74–1.50	0.77
Q3	1.45	1.05–1.99	0.02	1.45	1.05–2.01	0.02
Q4	2.04	1.50–2.78	<0.001	2.04	1.49–2.80	<0.001
Gn cycles						
EMT	1.00	0.93–1.08	0.94	0.98	0.91–1.06	0.65
Q1	*Ref.*			Ref.		
Q2	1.26	0.81–1.96	0.30	1.32	0.83–2.08	0.24
Q3	1.05	0.65–1.72	0.84	1.08	0.65–1.78	0.78
Q4	1.13	0.72–1.77	0.60	1.06	0.65–1.71	0.83
Ongoing pregnancy						
CC cycles ^†^						
EMT	1.10	1.05–1.16	<0.001	1.10	1.04–1.16	<0.001
Q1	*Ref.*			Ref.		
Q2	1.15	0.80–1.67	0.46	1.14	0.79–1.65	0.50
Q3	1.37	0.98–1.93	0.07	1.35	0.95–1.90	0.09
Q4	2.02	1.44–2.82	<0.001	1.98	1.41–2.79	<0.001
Gn cycles						
EMT	0.98	0.91–1.07	0.65	0.97	0.90–1.02	0.53
Q1	*Ref.*			Ref.		
Q2	1.19	0.75–1.87	0.46	1.27	0.79–2.03	0.32
Q3	0.88	0.52–1.48	0.63	0.92	0.54–1.57	0.75
Q4	0.99	0.62–1.59	0.97	0.95	0.58–1.57	0.85

CC, clomiphene; CI, confidence interval; EMT, endometrial thickness; Gn, gonadotropin; OR, odds ratio.

EMT was stratified by Quartiles (calculated separately for CC and Gn cycles). Generalized estimating equations were applied. EMT was assessed either as a continuous variable or by quartile increment.

*Adjusted for age, body mass index, prior gravidity, diagnosis, and day of last ultrasound.

^†^CC cycles included all cycles in CC1 and CC2.

## 4 Discussion

Our study investigated potential differences in endometrial thickness between CC/IUI and Gn/IUI cycles, and the impact these differences might have on IUI outcomes. In addition, we investigated differences in EMT between CC and Gn cycles associated with conception and those without. When patients were utilized as their own controls, our data suggested that the endometrium was significantly thinner in CC compared to Gn cycles, despite a clinically comparable follicular response. In late-follicular phase, a remarkable percentage of CC cycles (around 40%) had an EMT < 7 mm, a cut-off considered by many to negatively affect chances of clinical pregnancy (20). As expected, in CC cycles a thinner endometrium was associated with decreased CPR and OPR in our study population. However, no such association was observed in Gn cycles. This difference between CC and Gn cycles might be partially explained by the fact that the endometrium in Gn cycles was much thicker even in the lowest quartile. Another possible explanation is that the two medications may be impacting the endometrium differently, and in the case of CC through additional mechanisms that are not directly involved to the thickness of the endometrium.

Within the same patient, our results suggested that ovarian response, as assessed by total number of follicles ≥ 13 mm, was clinically similar between CC and Gn cycles, a finding that could be translated to comparable serum estrogen levels between regimens. However, CC stimulation still resulted in a much thinner late-follicular EMT than gonadotropins, which could provide further evidence for the anti-estrogenic effect of CC on the endometrium (21). Our results indicated that within the same patient, the EMT after gonadotropin is thicker than CC stimulation by an average of 1.7 mm. Similarly, Weiss et al. in a meta-analysis reported a thicker endometrium in Gn compared to CC cycles, but the difference appeared less prominent [mean 0.33 mm (95% CI: 0.01–0.64)] (4). Studies included in the meta-analysis differed in diagnoses of infertility (unexplained and mild male factor only) and study design (11, 22).

Although a thinner endometrium was found in women who conceived with CC (CC2) compared to those who switched to Gn (CC1), determining the clinical relevance of thin endometrium in the fertility setting remains challenging. While a clear cut-off defining “thin” endometrium does not exist, in most studies late-follicular phase endometrium measuring less than 7 mm or 8 mm is considered to be “thin” (14). In IVF cycles, where estrogen levels are much higher and the only ovarian stimulation medications used are gonadotropins, thin endometrium, defined as less than 7 mm, is rather rare and its reported incidence varies from 1% to 2.5% (20). However, relevant data is lacking in OS/IUI cycles. Our study showed that on the day of HCG, around 40% of CC cycles had a late-follicular EMT < 7mm, while in Gn cycles only 15% were below the same cut-off. Similarly, a recent RCT reported that 45% of CC cycles had EMT ≤ 7 mm among women with a history of six failed cycles (23).

Studies evaluating the impact of a thinner endometrium on pregnancy outcomes have been inconsistent, with a few reporting that it is associated with lower pregnancy rates (24, 25), while others not (4). A retrospective study reporting on a much smaller sample of CC/IUI cycles reported that pregnancy rates did not differ substantially between EMT strata and concluded that treatment decisions regarding switching from CC to other regimens should not be influenced by the thickness of the endometrium (26). On the contrary, a recent RCT on women with a history of six failed ovulatory CC cycles reported higher live birth rates when CC was switched to Gn among subjects with EMT ≤ 7 mm in the last CC cycle. No such benefit was reported for those who developed an “appropriately thick” endometrium with CC (EMT >7 mm) (23). In our study, pregnancies were observed even with endometria below the 5^th^ percentile (4.5 mm and 6 mm for CC and Gn, respectively), albeit at significantly lower rates. This finding suggests a negative, but not deleterious, impact of thin endometrium on CPR among CC cycles, which indicates that women developing a particularly thin endometrium following CC administration might benefit from switching to Gn.

Interestingly, in Gn cycles our data did not suggest an association between CPR and EMT. This finding is in agreement with a recent secondary analysis of the AMIGOS trial, showing no differences in EMTs between Gn/IUI cycles that led to live birth and those did not (18). Similarly, Liu et al. in a retrospective study also showed that EMT did not predict clinical pregnancy in Gn/IUI cycles (adjOR: 1.63, 95% CI: 0.71–3.77) (17).

The fact that a thinner endometrium negatively impacted CPR and OPR in CC cycles but not in Gn cycles suggests that the mechanisms mediating such action are not limited to the development of a thin endometrium but might involve additional factors. Hsu et al. reported that compared to unstimulated natural cycles, CC significantly decreased uterine blood flow during the early luteal phase, potentially impairing implantation and thus contributing to lower pregnancy rates (27). The significantly higher incidence of thin endometrium in CC cycles as compared to Gn and its potential effect on pregnancy rates suggests that the OS regimen should be considered when defining thin endometrium and when deciding how to manage IUI cycles affected by it. Additionally, evidence has indicated that Gn use is associated with increased risk of multiple pregnancies (8, 18), so once a decision is made to utilize Gn instead, appropriate counseling, stricter dosing regimens, careful monitoring, and IUI cancellation for over-response, should be considered to decrease risk of multiples.

To the best of our knowledge, our study was the first to evaluate EMT using patients as their own controls, with obvious benefit of minimizing the impact of potential confounders and allowing for the estimate of within-patient variability. The inclusion of cycle characteristics allows us to gain a better insight in the mechanisms responsible for the observed differences (e.g. follicular response being clinically comparable between CC and gonadotropin stimulation, in part because of the mild gonadotropin stimulation protocols used in our practice). In addition, laboratory and clinical protocols were consistent in all cycles since they were all conducted within one. However, several limitations should also be taken into consideration. First, selection bias could be introduced due to the retrospective nature of the study. Second, there might be possibility of residual confounding as information regarding other potential confounders such as estradiol level in the CC cycles, and lifestyle was not collected. Besides, endometrial pattern, which is often used to assess endometrial development, was not captured in this study. Third, although no association between EMT and CPR was found among Gn cycles in our study population, it should be noted that EMT was in an “acceptable” range even in the lowest quartile. Additionally, although letrozole use has increased over the recent years, in our database the percentage of patients that pursued gonadotropin IUI treatments after failing letrozole was relatively small (<5% of all IUI cycles included letrozole), limiting our power to investigate letrozole induced changes in EMT when utilizing patients as their own controls. Further prospective large scale cohort studies are still warranted to evaluate the impact of EMT on pregnancy and IUI outcomes among different OS regimens.

## 5 Conclusion

Our study showed that CC stimulation resulted in a thinner endometrium compared to Gn; and within-patient, the EMT was thinner in CC cycles by an average of 1.7 mm. Patients who conceived with CC had a thicker endometrium compared to those who failed and had to eventually pursue gonadotropin treatments. In CC cycles, a thinner endometrium was associated with decreased CPR and OPR, while in Gn cycles, no such association was observed. However, clinical implications of these findings and whether or not this should affect patient counseling is a topic for further discussion. Future research should focus on establishing the cut-off for thin endometrium among different ovulation stimulation regimens, and its impact on IUI outcomes.

## Data availability statement

The data analyzed in this study is subject to the following licenses/restrictions: The data underlying this article cannot be shared publicly to protect the privacy of individuals included in the study. Requests to access these datasets should be directed to isouter@mgh.harvard.edu.

## Ethics statement

The studies involving humans were approved by Partners Institutional Review Board. The studies were conducted in accordance with the local legislation and institutional requirements. Written informed consent for participation was not required from the participants or the participants’ legal guardians/next of kin in accordance with the national legislation and institutional requirements.

## Author contributions

YL: Conceptualization, Formal Analysis, Funding acquisition, Methodology, Visualization, Writing – original draft, Writing – review & editing. PC: Data curation, Investigation, Methodology, Validation, Writing – review & editing. VJ: Data curation, Investigation, Resources, Writing – review & editing. ID: Data curation, Investigation, Resources, Writing – review & editing. KJ: Methodology, Software, Validation, Writing – review & editing. CB: Investigation, Resources, Supervision, Writing – review & editing. IS: Conceptualization, Methodology, Project administration, Resources, Supervision, Writing – review & editing.
