# Supplementary material for: The impact of clomiphene citrate on the endometrium in comparison to gonadotropins in intrauterine insemination cycles: is it thinner and does it matter?
Source: Front Endocrinol (Lausanne). 2024 Jun 24;15:1414481. doi: 10.3389/fendo.2024.1414481 (PMC11228293; doi:10.3389/fendo.2024.1414481)
Supplement: Supplementary file 1 [file DataSheet_1.docx]

Supplementary Material

The impact of clomiphene citrate on the endometrium in comparison to gonadotropins in intrauterine insemination cycles: Is it thinner and does it matter?

Yao Lu, MD*, Panagiotis Cherouveim, MD, Victoria Jiang, MD, Irene Dimitriadis, MD, Kaitlyn E. James, PhD, Charles Bormann, PhD, Irene Souter, MD

*** Correspondence:** Yao Lu, MD, MMSc. Email: yaoluinrenji@163.com

## Supplementary Figure


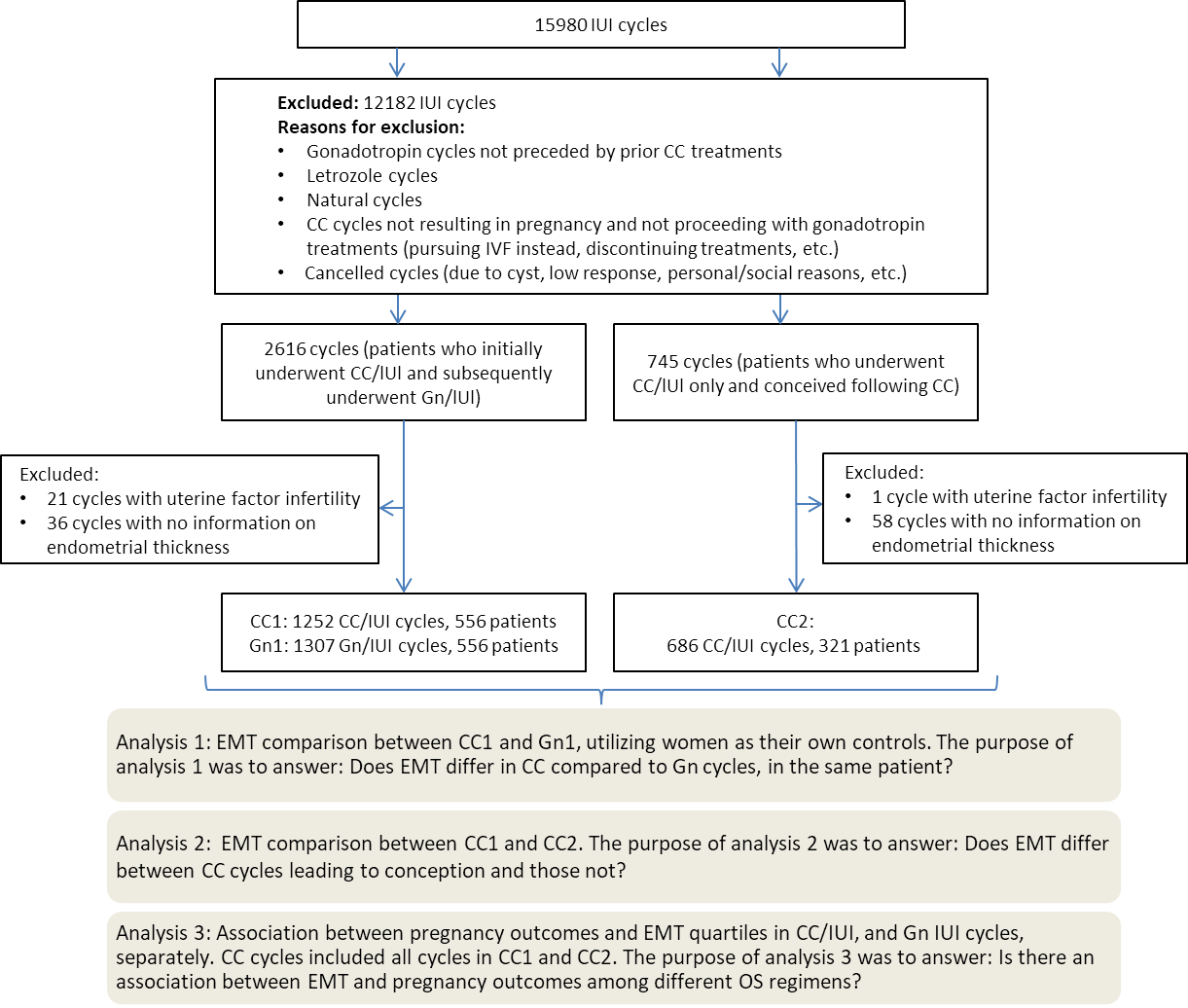


**Supplementary Figure 1.** Flow diagram of the study population. CC, clomiphene; EMT, endometrial thickness; Gn, gonadotropin; IUI, intrauterine insemination.
